# A novel approach to probe host-pathogen interactions of bovine digital dermatitis, a model of a complex polymicrobial infection

**DOI:** 10.1186/s12864-016-3341-7

**Published:** 2016-12-01

**Authors:** Paolo Marcatili, Martin W. Nielsen, Thomas Sicheritz-Pontén, Tim K. Jensen, Claus Schafer-Nielsen, Mette Boye, Morten Nielsen, Kirstine Klitgaard

**Affiliations:** 1 Center for Biological Sequence analysis, Technical University of Denmark, Kemitorvet, 2800 Kgs., Lyngby, Denmark; 2 National Veterinary Institute, Technical University of Denmark, Bülowsvej 27, 1870 Frederiksberg C, Denmark; 3 Schafer-N ApS, Lersø Parkallé 42, 2100 Copenhagen, Denmark; 4 Molecular Diagnostic and Clinical Research Unit, Hospital of Southern Jutland, 6400 Sønderborg, Denmark; 5 Instituto de Investigaciones Biotecnológicas, Universidad Nacional de San Martín, Buenos Aires, Argentina

**Keywords:** Integrated pipeline, RNAseq, Digital dermatitis, High-density peptide arrays

## Abstract

**Background:**

Polymicrobial infections represent a great challenge for the clarification of disease etiology and the development of comprehensive diagnostic or therapeutic tools, particularly for fastidious and difficult-to-cultivate bacteria. Using bovine digital dermatitis (DD) as a disease model, we introduce a novel strategy to study the pathogenesis of complex infections.

**Results:**

The strategy combines meta-transcriptomics with high-density peptide-microarray technology to screen for *in vivo*-expressed microbial genes and the host antibody response at the site of infection. Bacterial expression patterns supported the assumption that treponemes were the major DD pathogens but also indicated the active involvement of other phyla (primarily *Bacteroidetes*). Bacterial genes involved in chemotaxis, flagellar synthesis and protection against oxidative and acidic stress were among the major factors defining the disease.

**Conclusions:**

The extraordinary diversity observed in bacterial expression, antigens and host antibody responses between individual cows pointed toward microbial variability as a hallmark of DD. Persistence of infection and DD reinfection in the same individual is common; thus, high microbial diversity may undermine the host’s capacity to mount an efficient immune response and maintain immunological memory towards DD. The common antigenic markers identified here using a high-density peptide microarray address this issue and may be useful for future preventive measures against DD.

**Electronic supplementary material:**

The online version of this article (doi:10.1186/s12864-016-3341-7) contains supplementary material, which is available to authorized users.

## Background

Polymicrobial diseases are clinical and pathological manifestations induced by the presence of two or more microorganisms [1]. These infectious organisms act synergistically or in succession to mediate complex disease processes [2]. Examples of co-infections include periodontitis, otitis media, pneumonia and cystic fibrosis in humans, and bovine and porcine respiratory disease complexes, ovine foot rot and bovine digital dermatitis in livestock animals [2]. Many of these infections are serious diseases for which the etiological agents can be difficult to diagnose and treat. Novel approaches are needed to elucidate the mechanisms underlying pathogenesis and to prevent and treat these challenging disorders. In this study, we investigated bovine digital dermatitis (DD) both as a model of a complex polymicrobial infection and as a disease in its own right.

DD is one of the major causes of lameness in cattle and constitutes an increasing problem in modern dairy farming worldwide [3, 4]. This inflammatory skin disease is characterized by focal proliferative to ulcerative dermatitis typically located on the plantar aspect of the hoof [5]. Present treatments include topical antibiotics and footbaths, but DD is difficult to eliminate, and infections are often recurrent [3, 6]. Recently, culture-independent microbial community profiling using 16S rRNA gene analysis revealed a high prevalence of invasive treponemes with genetic heterogeneity in DD lesions [7–11]. Other taxa identified from DD lesions by bacteriological, histopathological, and molecular biological investigations include *Porphyromonas*, *Prevotella*, *Fusobacterium*, *Campylobacter*, *Bacteroides*, *Mycobacterium, Mycoplasma,* and *Guggenheimella* [10, 12–14].

Our understanding of the *in situ* activities of community microorganisms and their mutual interactions with the host remains limited despite increased knowledge of the phylogenetic composition of the DD microbiome. Similarly, there is a lack of information concerning efficacious immunoprophylactic antigens against DD. Cows exhibit both humoral and cellular immune responses to DD-associated treponemes [15] and possibly other bacterial groups [13]. However, the variable host humoral response against different isolates of *Treponema* strongly indicates that DD-associated treponemes possess considerable antigenic variation [16–18]. Because of these antigenic variations, combinations of antigens from multiple *Treponema* species and other bacterial taxa possibly involved in the development of infection should be used for serological analysis and the development of disease prevention measures [18]. At present, identification of these antigens is hampered by the lack of methods to isolate treponemes from DD lesions. To address this challenge, we used meta-transcriptomic analysis to characterize the *in situ* gene expression patterns of the microbiome in DD-affected skin lesions and the host antibody response directed at the site of infection. We report the first *in situ* genome-wide transcriptome study of the microbiome in DD-affected skin lesions from 21 dairy cows. From the transcriptome data, we identified a panel of *Treponema* genes that were highly expressed in multiple animals, and we monitored the host immune response to these target genes using high-density peptide microarrays. Using this approach, we identified a number of potential virulence factors and immune modulators that represent the environmental stimuli encountered during infection. Furthermore, the microbial gene expression profiles enhanced our understanding of the core activities characteristic of DD and the role that individual organisms in the infected hoof play in the development of disease.

## Methods

### Biopsy and blood sample collection and serum purification

A total of 21 dairy cattle (Danish Holstein breed) were sampled in four dairy herds located in geographically diverse regions of Denmark. Herds were selected due to their recurrent history of DD and the respective herds ranged from 100-301 lactating cows - all kept in loose housing systems. In each herd, skin biopsies were collected from 4-6 animals with active ulcerative DD lesions. Before sampling, the lesion surface was gently cleaned with water, and the area was locally anesthetized. Biopsies were taken from the center of the lesion with a 6 mm sterile punch biopsy needle (KRUUSE Group, Denmark) and immediately transferred to a nucleic acid stabilization solution (RNA*later®*, Ambion, Austin, TX, USA).

Blood samples for serum collection (2 x 8.5 ml/cow) were taken from the jugular vein using vacuum collection tubes containing a clot activator additive (BD Vacutainer®, BD SST™, BD Hemogard™, Becton Dickinson, Franklin Lakes, NJ, USA). A total of 23-25 blood samples were collected from each of the four herds. The blood samples were taken from the biopsied cows and randomly selected animals with unknown lesion status. Additional blood samples were collected from Danish Holstein cows in dairy herds with no clinical history of DD. Serum was purified by centrifugation 2000 x g for 10 minutes. The individual serum fractions were subsequently stored at -80 °C until analysis. All biopsies and blood samples were collected in accordance with the Danish Animal Experiments Inspectorate under the Ministry of Justice.

For peptide chip screening, individual serum fractions were pooled in a 1:1 volumetric manner to generate a DD-positive and DD-negative pool. All defrosted serum pools were supplemented with 0.1 μg/μL sodium azide (Merck Millipore, Darmstadt, Germany).

### Total RNA isolation and sequencing

**Fig. 1:**
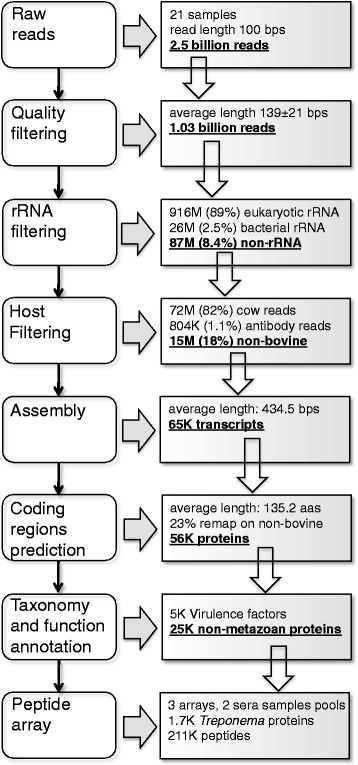
Bioinformatics pipeline for RNA-seq data filtering and assembly. In each box, the red text indicates the data used in the following step of the pipeline

Total RNA was isolated from 20-30 mg of biopsy tissue material. Minced tissue was transferred to a tube containing lysis buffer provided with the RNeasy Fibrous Tissue Mini Kit (Qiagen, Hilden, Germany) supplemented with β-mercaptoethanol (Sigma Aldrich) according to the manufacturer’s instructions. Tissue samples were disrupted with a stainless steel ball in a Tissue Lyser II (Qiagen, 2 x 2 min at 20 Hz). The remaining RNA extraction steps were performed according to the supplier’s protocols. An additional DNA elimination step was performed with TURBO™ DNase following the manufacturer’s protocol (Ambion, Cambridgeshire, UK). RNA concentrations and quality were measured using a NanoDrop ND-1000 (Thermo Scientific, Wilmington, DE, USA) and an Agilent 2100 Bioanalyzer (Agilent Technologies, Santa Clara, CA, USA), respectively. All preparations used in this study had the following values: 260/A280 ≥ 1.8, OD260/230 ≥ 2.0 and RIN ≥7.0. All RNA samples were stored at -80 °C and shipped with dry ice. RNA libraries were prepared using a strand-specific protocol, and paired-end reads (read length 100 bp and insert length 100-500 bp) were sequenced using the Illumina Hiseq2000 platform (San Diego, CA, USA). The pipeline used to filter and assemble the RNA-seq data into transcripts is described in Fig. 1. Additional details on the procedure are presented in the Additional file 1: Supplementary Materials, and resulting assembled transcript sequences are presented in the Additional file 2: Supplementary Materials.

### Identification of bovine immunoglobulin reads

All non-ribosomal reads were mapped to a library containing the *Bos taurus* germline V, D and J genes [19, 20] (courtesy of authors Antti Livanainen and Ulrike S. Diesterbeck), and mature antibody sequences were retrieved from the DIGIT database [21] using TopHat2 software with relaxed parameters allowing for up to 30 mismatches, 50 gaps, and both should sum up to an overall edit distance of no more than 60. This was done to take into account somatic mutations and possible missing germ lines in the currently sequenced bovine antibody gene repertoire.

### Transcript annotation

When possible, up to three annotations were assigned to each transcript as follows: a taxonomical, functional, and virulence annotation.

For the taxonomical annotation, the UniProt database was scanned with all transcripts using the Blastx software. To filter out possible host or external contaminants not detected in the previous steps, all transcripts with a best hit (in terms of Blastx e-values) to a Metazoan transcript were discarded from the subsequent analysis. This filtered dataset consisted of 26,634 transcripts with an average length of 125.9 residues. Next, every transcript with a positive hit (e-value <10e-5) with at least 50% coverage was annotated with the hit family (if sequence identities were at least 60%) and species definition (if sequence identities were at least 95%) if present.

Each transcript was blasted in the following databases: Clusters of Orthologous Groups of proteins (COGs) [22], VFDB (http://www.mgc.ac.cn/VFs), Victors (http://www.phidias.us/victors), and PATRIC VF [23]. Only hits with e-values <10e-5 were considered. For the functional annotation, transcripts were assigned to the Id and Class of each COG hit. Finally, transcripts with at least one hit on Victors, VFDB, and PATRIC VF were annotated as virulence factors.

### Expression analysis

The abundance of each transcript in the filtered dataset, measured as the number of mapped reads and as fragments per kilobase of transcript per million mapped reads (FPKM), was calculated by mapping the reads obtained after removing the bovine transcripts. We defined the abundance of a given taxonomic unit in a sample as the sum of reads in the sample that map on all the transcript that had been assigned to that specific taxonomic unit (see previous section for details). By focusing on transcripts that were expressed in 11 or more samples, we defined high expression core (HEC) and low expression core (LEC) genes as transcripts with an average FPMK above or below 46.8 (average value of transcript expression calculated only for samples in which the transcript was expressed), respectively.

### Assembly-independent abundance

We used a k-mer (sequence fragments of length *k*)-based count to estimate the variability in the bacterial transcriptome and the antibody response in each sample. Because the amounts of bacterial and antibody sequences varied considerably between samples, we used a subsampling/bootstrapping strategy. In order to avoid biases due to the different sequencing depth and bacterial load, a sub-sample of 6 000 reads mapping to bacterial transcripts or antibodies was randomly extracted 10 times; each time, 500 000 15-mers were randomly selected from the set. We defined the diversity of the bacterial and antibody repertoire as the average proportion of different k-mers observed in each 500 000 k-mer selection.

### Peptide array design and analysis

From the overall set of transcripts, we selected those annotated as treponemal that were expressed in at least 7 different samples and that had an average expression of at least 36 FPKM. We also selected all treponemal transcripts that were expressed in at least 4 samples with an FPKM of at least 15 and were predicted to contain either a signal peptide or at least one transmembrane helix [24, 25]. As a control of our *de novo* protocol for the identification of potential antigens, and given the lack of information on known or putative virulence factors in DD, we included in this list a set putative virulence factors of the human oral pathogen *Treponema denticola* previously described in the literature [26, 27] and their homologous proteins in *Treponema phagedenis* (Additional file 3: Table S1). Indeed, 29 out of 51 of these proteins were either identical or with high sequence similarity (80% identity or more) to transcripts found in our sequencing data.

Then, we extracted all 15-mer peptides present in this protein set to obtain a final library of 194 295 peptides. Three identical peptide arrays were designed using this peptide list.

**Fig. 2:**
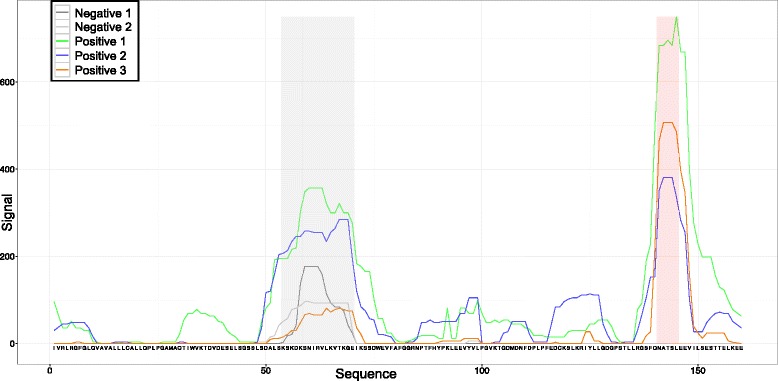
Peptide array signals for a transcript. Red and gray areas indicate specific and non-specific regions, respectively

Two arrays were screened with a pool of sera from healthy cows from naïve herds with no endemic DD, and the intensity of the signal for each peptide was measured. We refer to these experiments as “negative chips”. All three arrays were subsequently screened with a pool of sera from DD-afflicted cows/herds, and the intensity of the signal was measured for each peptide. We refer to these experiments as “positive chips.” The peptide intensities were mapped back onto the original transcripts by constructing transcript-specific reactivity profiles. We used the average when more than a single peptide mapped at the same position on a transcript. Missing values were replaced by zeros. Then, a running median filter was applied to the sequences by assigning to each peptide the median intensity value of the peptides centered 2 residues before through 2 residues after its position. Finally, all sequence profiles were scanned to detect peaks on the negative chips (non-specific peaks) using an intensity threshold equal to the 90^th^ percentile of all intensities on the corresponding negative chip. If a peak was detected on any negative chip, a region of size 3 centered on the peak was used to define the non-specific regions in the transcripts (Fig. 2).

The chip data were used to assign an immunogenicity score to each transcript. We transformed all signals in the positive arrays to Z-scores by subtracting the chip average signal and dividing by the standard deviation. Then, we assigned a score to each peptide in each transcript equal to the average Z-scores of the 3 positive arrays. Finally, each transcript was assigned a *raw immunogenicity score* equal to the maximum of all such peptide scores, and a *filtered immunogenicity score* was obtained equal to the maximum of all peptide scores excluding the non-specific regions.

## Results

### Phylogenetic analysis of the metatranscriptome

**Fig. 3:**
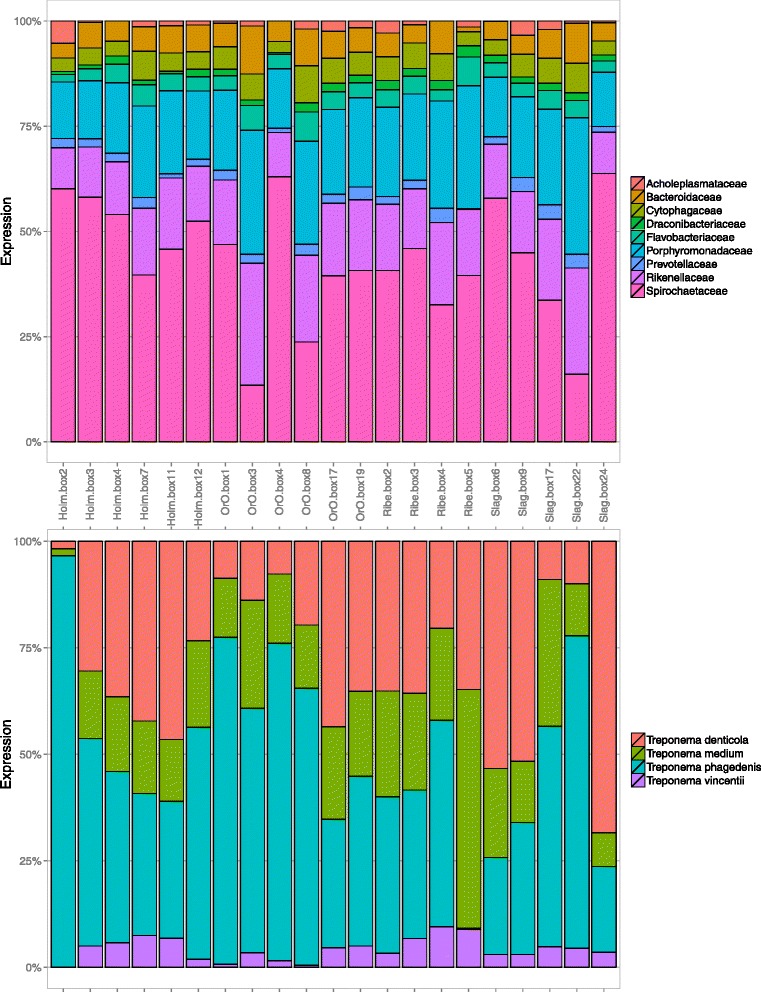
Taxonomic composition of the samples. The family (**a**) and species (**b**) compositions of the bacterial transcripts identified in the samples. The expression has been estimated as the sum of the reads mapped on all transcripts annotated with a given taxonomic unit. Unmapped reads and transcripts with no taxonomic annotation are not displayed

Transcripts from total RNA extractions of 21 biopsies originating from 4 different dairy herds were sequenced and mapped against Uniprot by applying the procedure outlined in the Materials and Methods (see Fig. 1). The phylogenetic distribution derived from the transcripts is shown in Fig. 3.

The microbiota transcripts with the highest expression levels in the DD lesions belonged to the phyla *Spirochaetes*, *Bacteroidetes* and *Firmicutes*. At the family level, *Spirochaetaceae*, *Porphyromonadaceae*, *Rikenellaceae,* and *Bacteroidaceae* had the highest numerical distributions (Fig. 3a). This population structure diverged considerably from the microbiota of healthy intact cow skin, which is usually dominated by *Moraxellaceae*, *Corynebacteriaceae*, *Lactospiraceae,* and *Ruminococcaceae* [10, 11]. Within the genus *Treponema*, the identifiable transcripts were primarily assigned to *T. denticola*-like, *T. phagedenis*-like, and *T. medium*-like species (Fig. 3b).

A large variability in the microbiota composition (as measured in terms of taxa prevalence and/or taxa expression) was observed between samples both at the family and species levels. This variability was preserved even when we focused solely on transcripts that were conserved across different samples, imposed by large variations in expression levels. We classified the transcripts into three groups depending on their expression levels and prevalence in the different samples: highly expressed core (HEC) genes (5.6%), lowly expressed core (LEC) genes (5.6%), and non-core genes (88.8%) (see the Materials and Methods for details). *Bacteroidetes* and *Spirochaetes* were the dominant phyla in the highly expressed core (HEC) genes. At the genus level, *Bacteroidetes* core genes were primarily composed of the genera *Alistipes*, *Odoribacter*, *Bacteroides*, *Cytophaga,* and *Porphyromonas,* most of which are ubiquitously found in cattle feces [28] (Additional file 1: Figure S1). All of the spirochaetes belonged to the genus *Treponema*, with *T. denticola*, *T. phagedenis, T. medium* and *T. vincentii* as the most abundant species.

### Expression of putative virulence factors in the microbial community of DD lesions

A total of 536 putative virulence factors (PVFs) were identified in the 1947 HEC genes. The phyla *Bacteroidetes* (primarily the genera *Alistipes*, *Bacteroides*, *Odoribacter,* and *Porphyromonas*) and *Spirochaetes* (solely the genus *Treponema* spp.) expressed approximately 50% and 25% of the putative PVFs, respectively. The remaining 25% mostly originated from the phyla *Firmicutes* and *Tennericutes*.

**Fig. 4:**
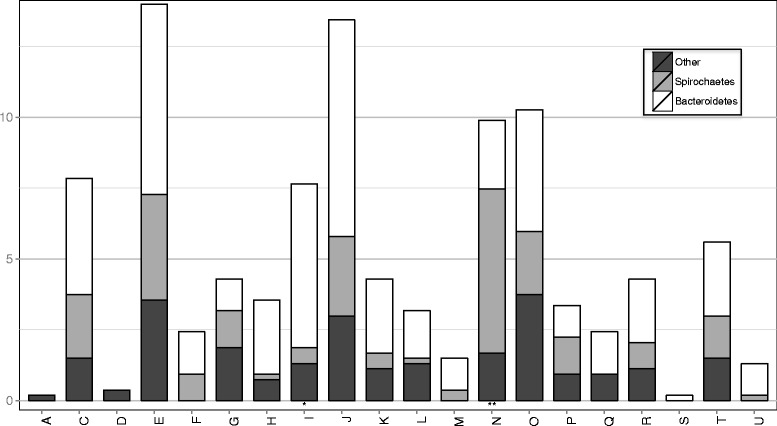
Amount of predicted virulence factors grouped by their COG class. One or two asterisks indicate the classes in which a statistically significant enrichment or depletion of Spirochaetes virulence factors is observed (chi-square test) with a *p*-value less than 0.05 or 0.001, respectively

The bacterial PVFs were classified according to their COG (Clusters of Orthologous Group) terms (Fig. 4). A considerable number of the PVFs (35%) was annotated as being involved in the transport and metabolism of amino acids (E; 14%), lipids (I; 7%), coenzymes (H; 4%), carbohydrates (G; 3%), nucleotides (F; 3%), and inorganic ions (P; 3%). Among the genes included in these COGs were ABC transporters engaged in the transport and metabolism of amino acids, inorganic ions and carbon hydrates. Interestingly, *Treponema* was the only genus to express Na^+^-dependent symporters (alanine, proline and glutamate), which are driven by sodium motive forces (SMFs). We also observed a putative Na^+^-transporting methylmalonyl-CoA/oxaloacetate decarboxylase (*oadB*) among the *Treponema* PVFs, which seemed to indicate that the DD-associated treponemes relied on the Na^+^ cycle for at least part of their energy metabolism.

Genes of the arginine deiminase (ADI) pathway, such as arginine deiminase (*arcA*), ornithine carbamoyltransferase (*arcB*), and carbamate kinase (*arcC*), were identified from the HEC PVFs of *Treponema* and *Odoribacter*. Both *T. denticola* and *T. phagedenis* use arginine as an energy source [29, 30]. The ADI pathway is also a major defense mechanism against acid stress [31]. Genes encoding enzymes involved in the catabolism of glutamate (glutamate dehydrogenase) and glycine (glycine cleavage system proteins) were also present among the PVFs of both *Spirochaetes* and *Bacteroidetes*. Among the genes involved in inorganic ion transport, a potential DNA-binding ferritin-like protein (Dsp) was expressed by both members of the genera *Treponema* and *Porphyromonas*. Dsp is found in many bacterial species and protects cells from nutritional deprivation or oxidative stress by sequestering iron and limiting Fenton-catalyzed oxyradical formation [32]. In *T. pallidum*, a Dsp-like protein (TpF1) may play a central role in both the early-stage inflammatory process and long-term persistence within the host [33].

Another functional group over-represented in the HEC PVFs of both *Spirochaetes* and *Bacteroidetes* was “post-translational modifications/chaperones” (O; 10%). This COG includes the chaperones/heat shock proteins (HSPs) encoded by *groEL* (HSP60 family) and *groES* (HSP10) and the peroxiredoxin (*ahpC*), thioredoxin reductase (*trxR*) and thioredoxin (*trx*) genes. The latter three genes encode proteins that protect the bacteria against hydrogen peroxide and superoxide, which are two of the principal reactive oxygen species (ROS) that bacteria must protect themselves against in the host [34]. The Prx-Trx peroxide defense system is a major source of protection against oxidative stress in *T. pallidum* [35].

Putative genes from the COG energy production and conversion (C) term constituted approximately 10% of the PVFs. From both *Bacteroidetes* and *Spirochaetes*, this functional class comprised important enzymes associated with the fermentation of carbohydrates under anaerobic conditions, such as the pyruvate:ferredoxin oxidoreductase (PFOR) and pyruvate-formate lyase (PFL).

Finally, motility and chemotaxis genes were amply represented among the putative PVFs (N; 7%). These PVFs included genes predicted to encode filament core proteins (*flaB*), basal body, rod and hook proteins (*flgL*), and motor switch proteins (fliM). A putative chemotaxis response regulator containing a CheY-like receiver domain was also among the PVFs. More than half of these genes originated from the treponemes.

### Diversity of the bacterial transcriptomes between samples

**Fig. 5:**
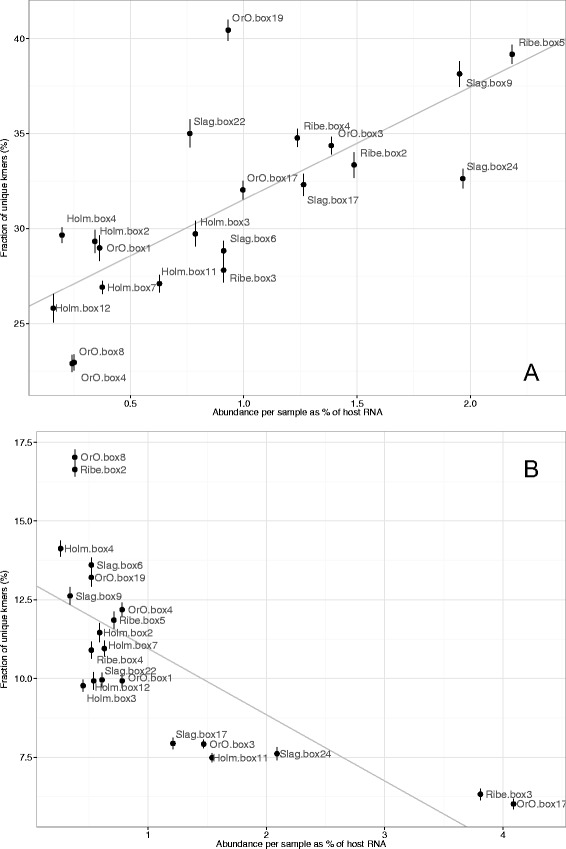
Diversity vs abundance in the samples. Abundance is calculated as the ratio between reads that map on the bacterial (panel **a**) and antibody (panel **b**) transcripts and the reads that map on the cow genome. Diversity is defined in panels **a** and **b** as the average percentage of unique k-mers for every 500 000 k-mers extracted from the bacterial and antibody reads. Regression lines are displayed in grey (panel **a** Pearson correlation = 0.75, *p*-value = 9.1e-5; panel **b** Pearson correlation = -0.73, *p*-value = 0.0017). Bars indicate ±1 standard deviation, calculated over 10 k-mers extractions

We observed very limited sample-wise conservation among the transcripts identified in this study. Only ~8% of the putative bacterial proteins were in the HEC group, and among these, only 25% showed similarity to known virulence factors. The taxonomical annotation provided in this study described only the most expressed species and those for which an annotated and highly similar entry in the Uniprot database could be found, thereby excluding all putative bacterial reads that could not be properly assembled into transcripts or that shared less than 60% sequence identity to any protein in Uniprot. To avoid these biases imposed by transcript assembly and annotation and to quantify the diversity of the bacterial RNA material in each sample, we used the assembly-independent k-mer-based estimate described in the Materials and Methods. We observed a strong correlation between bacterial diversity and abundance (Fig. 5a). The diversity of the bacterial transcriptomes varied extensively between different samples, ranging from 21% to 41% unique k-mers for every 500 000 randomly extracted k-mers. This variability was not correlated to the geographical source of the samples or the presence of a dominant species (Additional file 1: Figure S1). For example, although the Slag Box24 sample was clearly dominated by *T. denticola* (see Fig. 3), it displayed large bacterial variability in k-mers.

### Antibody sequence analysis

We used the same k-mer-based approach to provide a global description of the host humoral immune response. The antibody diversity in the samples (defined as the proportion of unique k-mers in antibody reads) was generally extremely large and variable, ranging from 6% to close to 17% of the unique k-mers. Note, that the constant part constitutes approximately 70% of antibody sequence, and exhibit very limited variability, and diversity in a response consisting of a single IgG clone would be as low as 0.4% of unique k-mers. Interestingly, the samples with the lowest diversity (lowest ratio of unique k-mers) were those in which the normalized antibody abundance, calculated as the ratio between the number of antibody reads and the overall amount of bovine reads, was highest (Fig. 5b).

We did not observe a correlation between the abundance and diversity of the antibody repertoire in the samples with the bacterial diversity (Additional file 1: Figure S2).

### High-density peptide microarrays

In agreement with earlier peptide array experiments [36], a high correlation was observed between the signals from the positive and negative arrays, thereby confirming that only a minor fraction of the antibody responses was DD-specific. There was a significant correlation between the raw immunogenicity score and the number of samples in which a given protein was observed (data not shown). A smaller yet still significant correlation was observed between the filtered immunogenicity scores and conservation across samples. By contrast, no correlation was observed between the immunogenicity scores and overall transcript expression (Additional file 1: Figure S2).

Notably, only few of the antigens identified using the peptide arrays were observed in all samples with high expression levels. This result is most likely connected to the extreme variability of the pathogens’ expression patterns observed in the samples and can explain the inability of the host immune system to prevent subsequent re-infection.

The 133 transcripts with a filtered immunogenicity score larger than 0.7 are reported in Additional file 4: Table S2. They include a number of known antigenic proteins/virulence factors, such as flagella proteins, cytoplasmic filament protein A, factor H binding protein (fHbp), transporter proteins and major surface antigen (Msp) identified in *Treponema* spp. [27, 37].

### Antigenic potential of the most highly expressed *Treponema* genes during infection

By combining the transcriptomics data with the results of the peptide array, we obtained an overview of the antigenic potential of thousands of proteins. Many proteins that were identified as immunogenic were not expressed or were expressed with low abundance in more than half of the analyzed samples. Some of the most immunogenic transcripts among the HEC genes primarily encoded proteins of unknown function. Together, the identified genes, represented potential new targets for therapeutic intervention. For example, the glycolytic enzymes glyceraldehyde-3-phosphate dehydrogenase (GAPDH) and enolase were expressed in 67% of the animals. These virulence-associated immunomodulatory proteins have other properties in addition to glycolysis and can translocate to the cell surface, where they play important roles in host-pathogen interactions [38–40]. Furthermore, both enzymes appear to have protective antigenic potential [40–42]. Other proteins with possible antigenic potential are the periplasmic component of the ABC-type dipeptide transport system and a potential Thioredoxin protein (Additional file 4: Table S2).

Interestingly, many of the flagellar genes were highly immunogenic in both DD-affected and healthy control cows. The flagellar proteins appear to be conserved among species of *Treponema* [43], and strong cross-reacting epitopes [44] may be shared with environmental spirochetes encountered in tissues, such as the bovine rumen. This phenomenon has been observed for another spirochete, *Borrelia burgdorferi* [45].

## Discussion

To gain insight into the *in situ* activities of microorganisms associated with DD and the host antibody response to infection, we combined a meta-transcriptomic analysis with high-density peptide microarray technology. This approach allowed us to target the bacterial community members most likely to be involved in disease development and to delineate common molecular virulence determinants that could enable the invasion and persistence of bacteria in the host. Furthermore, we measured the antigenic potential of the most highly expressed *Treponema* genes during infection and estimated the repertoire of the antibody response elicited by the bovine host.

### Taxonomic composition of the infecting microbiota

To date, phylogenetic characterizations of the DD microbiome have been derived from deep sequencing analyses of 16S rRNA genes, fluorescence *in situ* hybridization and culturing [10, 46, 47]. In the present study, the contribution of individual members of the DD microbial communities to disease development was estimated from the inferred taxonomy of the expressed gene profiles of bacteria isolated from the site of infection. When measured on the raw reads and final assemblies, the variability of the bacterial meta-transcriptomes expressed in the individual cows was surprisingly high, with only 5% of the putative transcripts highly expressed in more than 50% of the samples. This large variability could not be trivially explained by the geographical location of the cows from which the samples were obtained.

However, the taxa identified from the metagenomics expression profiles of the HEC genes accentuated the importance of *Treponema* spp. in the pathogenesis of DD. The data also indicated that opportunistic pathogens from the phylum *Bacteroidetes* (*Porphyromonas, Odoribacter*, *Cytophaga*, *Bacteroides,* and *Allistipes*) play a role in disease development. Of these, only *Bacteroides,* and *Porphyromonas* were previously associated with DD [11]. *Bacteroides* belongs to the group of commensals found in the intestinal tract of humans and animals (mouth, colon, and urogenital tract) that can switch to become opportunistic pathogens [48]. In combination with other facultative/strict anaerobes, these bacteria can cause localized abscesses in many different tissues, including dental abscesses [49]. *Treponema*, *Porphyromonas,* and *Odoribacter* have also been associated with periodontal disease [50–52]. Therefore, our study supports previous observations of similarities between these two polymicrobial infections.

Consistent with previous investigations [8, 10, 11], we identified *T. phagedenis*-like, *T. medium*-like and *T. denticola*-like species as important pathogens of DD. Although the sequence identification at the species level is less certain, all *Treponema* species identified in this study were previously identified as major constituents of DD lesions by other methods [8, 53, 54]. Previous metagenomic studies, mainly based on the analysis of the 16S rRNA gene, have suggested that up to 20 different phylotypes may be involved in the pathogenesis of DD [8, 10, 47]. Unlike these studies, we only identified a few *Treponema* species. As the transcripts in our study were assembled *de novo*, we do not expect this to be an effect of the lack of template information due to the fact that many of these phylotypes have not yet been cultivated and sequenced. More likely the observed discrepancy is due to differences in expression levels – i.e. even though different phylotypes might been present, the expressed transcripts come from a minority of the bacteria.

The importance of other species previously identified from DD lesions by 16S rRNA gene analysis, such as *Fusobacterium necrophorum*, *Dichelobacter nodosus,* and *Mycoplasma fermentans* [10, 47], was not supported in the present study. However, these taxa could participate in the pathogenesis at other DD stages.

### Core virulence factors

Analysis of the potential PVFs of the highly expressed core (HEC) genes provided valuable information on the strategy adopted by the bacteria to survive and persist in the hostile surroundings within the host. The ability to move and respond chemotactically to environmental stimuli appears to be important for the DD microbiota and in particular the treponemes because known spirochetal PVFs with high representation among HEC genes are primarily related to these functions. This overrepresentation of proteins involved in chemotaxis and flagellar biosynthesis during infection was previously described for both *T. pallidum* and *T. denticola* [55, 56]. Otherwise, we detected relatively few of the classical spirochetal virulence attributes, such as *Msp*, hemin-binding protein B, hemolysins and leucine-rich repeats, among the HEC [27, 37]. Because many of the spirochetal PVFs have been identified from human pathogens from widely differing ecological niches, they may not be directly comparable with the unique functions or virulence attributes of the DD-associated treponemes [27, 30].

The number of identified PVFs engaged in metabolic processes and transport illustrate that biosynthesis and nutrient acquisition during infection are integral to pathogenesis. For instance, arginine, glutamate and glycine may be important sources of energy and metabolites for the infecting microbiota. This finding correlates well with the preference of *T. denticola* and *T. phagedenis* for amino acids instead of sugar as their primary energy source [30, 37]. The observed expression of Na^+^-dependent symporters and *oadB* indicated that some of the bacteria (primarily *Treponema* spp.) might use sodium ion cycles for energy generation. Sodium ion cycles appear to be common among human and animal pathogens and may provide adaptive advantages for these bacteria because they provide additional means of ATP synthesis, motility and solute uptake [57]. For example, *T. pallidum* energetics is exclusively based on SMF, which is generated by its Na^+^-motive oxaloacetate decarboxylase [57]. Another priority of the infecting microbiota was protection against host immune factors because we observed several genes involved in the stress response among the HEC PVFs. These included the chaperones *GroEL* and *GroES*; the ADI pathway, which is a major defense mechanism against acidic stress; a gene encoding Dsp; and the thioredoxin genes *aphC*, *trxR,* and *trx*, which all protect the bacteria against ROS [34, 58].

The samples used in the present study were collected from active, persistent DD lesions; thus, the overall picture of metabolic activity of the PVFs with relatively few classical PVFs and a prioritization of genes involved in anaerobic metabolic functions, chemotaxis, motility and stress are consistent with an adapted phenotype capable of persisting in the host environment [59]. Many of the virulence properties described here were observed in both *Spirochaetes* and *Bacteroidetes*, indicating that both phyla are part of the disease etiology. However, the treponemes appear to drive a large portion of the activity as measured by their gene expression in the DD lesions. Histological studies have previously shown that treponemes are prevalent in the deep parts of lesions, whereas other taxa are primarily located in the superficial parts of lesions [47, 53]. Although treponemes are most likely the primary pathogens, other bacterial phyla may still be of vital importance to pathogenesis (e.g., by facilitating skin colonization and lesion development) [47].

### Antibody response elicited by the bovine host

Cattle with DD develop high levels of antibodies against *Treponema* and other bacteria soon after infection occurs, but these antibodies do not offer protection against lesion development [18, 60, 61]. Here, we observed a large diversity in the repertoire of antibodies expressed in different cows with very small overlaps between the samples and signs of a focused clonal amplification in only 2 samples. This could possibly be due to different progression of infection in samples in which the immune response was at its peak or already regressing.

Surprisingly, this diversity showed limited correlation to the overall diversity of the bacterial transcriptome. We observed a large variability of pathogens in the lesions and the presence of a large number of different antigens, few of which were consistently found in all samples, which altogether might prevent an effective antibody response. This type of deceptive strategy is often observed in infectious diseases that are either polymicrobial [62] or caused by a single pathogen [63].

To further characterize the antibody immune response to DD, we analyzed the reactivity between cow antibodies and peptides derived from a subset of potentially antigenic proteins using high-density peptide arrays. Here, the antibody response was found to rarely targeted proteins that were highly expressed in most of the cows but was often directed at proteins that were found in only a few samples or at low abundance.

Our results support the hypothesis that the multimicrobial nature of the disease impairs the bovine immune system by offering a large number of secondary epitopes that function as decoys and prevent an effective immune response. This hypothesis would be consistent with the high number of reinfections observed in digital dermatitis and in the lack of efficacy of vaccines based on attenuated or killed bacteria cultivated *in vitro* to date [64]. A large number of different microbes can colonize the lesions, and the apparently highly diverse bacterial population can cause similar phenotypic effects. Even if we restrict our analysis to only *Treponema* transcripts, we observed a large diversity in the expression profiles in different samples.

## Conclusions

To the best of our knowledge, this study presents a novel methodology that combines RNA-seq and peptide array technology for the analysis of host-pathogen interactions at the site of infection. This approach can overcome some of the difficulties that presently impair the study of complex microbial infections. The data presented here show that the expression profile of each single pathogen involved in DD infection can vary tremendously between samples displaying the same pathology and that most antigens are expressed in only a few samples. In cases like this, the immunogenic potential of infectious targets can only be properly elucidated by combining *in situ* expression patterns and the host immunological response. The method we propose cannot, and should not, be used to identify the specific complex mechanisms that occur in single individuals, but rather to provide a global representation of the pathogenic mechanisms consistently observed in the infected samples. In this study, we identified a sub-set of antigenic proteins, which were expressed in the majority of the samples, and demonstrated antigenicity when screened against sera from infected animal. Further studies are needed to determine whether these proteins represent candidates for the development of novel biomarkers or vaccines.

## Additional files

Additional file 1: Supplementary Materials and Methods, Figure S1 (Taxonomic composition of the samples), Figure S2 (Bacterial vs. Antibody k-mer diversity), Table S3. (DOCX 163 kb)

Additional file 2: Transcript sequences. (TXT 6650 kb)

Additional file 3: Table S1. Known virulence factors in *T. denticola* and *T. phagedenis. (DOCX 125 kb)*

Additional file 4: Table S2. Immunogenicity scores. (XLSX 5486 kb)
