# Supplementary material for: A novel approach to probe host-pathogen interactions of bovine digital dermatitis, a model of a complex polymicrobial infection
Source: BMC Genomics. 2016 Dec 1;17:987. doi: 10.1186/s12864-016-3341-7 (PMC5142292; doi:10.1186/s12864-016-3341-7)
Supplement: Supplementary file 1 — Supplementary Materials and Methods, Figure S1 (Taxonomic composition of the samples), Figure S2 (Bacterial vs. Antibody k-mer diversity), Table S3. (DOCX 163 kb) [file 12864_2016_3341_MOESM1_ESM.docx]

**Supplementary Information**

**Materials and Methods**

*Illumina RNA Sequencing*

The RNA samples were submitted to the BGI Tech Solutions Co. Sequencing Centre, for sequencing by the Illumina high-throughput platform by BGI Tech RNA-seq (transcriptome) procedures, with the initial fragmentation at RNA level before cDNA synthesis in the library construction protocol. For each of the 21 samples, we sequenced on average 50 million (see supplementary table S3) pairs of strand-specific 100-base Illumina reads.

*Preprocessing and rRNA filtering*

Sequences were analyzed for quality using the FastQC software (version 0.9.0) (Andrews 2011). Any sequence overlapping with more than 3 bases at the 3’ end with the adaptors sequences was trimmed using the software cutadapt (version 1.2.1) (Martin 2011) and each read was trimmed by 10 bp at its 5’ end. Left and right reads overlapping with at least 10 bp were merged in a single-end read (flash version 1.2) (Magoc and Salzberg 2011). Both the single-end reads and the remaining of the paired-end reads that were not previously merged were subsequently trimmed at their 3’ using a quality threshold score (phred64 quality score) of 10. Finally, sequences shorter than 25 bps and sequences with a mean quality score lower than 20 were discarded.

*rRNA and bovine reads filtering, and final assembly*

Both the single- and the paired-end reads were mapped against the SILVA database of rRNA sequences (Quast *et al.* 2013) using the sortmerna software (version 1.99-beta) (Kopylova *et al.* 2012) with default settings. In a first round, reads that mapped against eukaryotic rRNA sequences were removed; the remaining reads were mapped against bacterial rRNA (supplementary table S3). All reads mapping to either bacterial or eukaryotic rRNA were discarded from further analysis. Figure S1 shows an overview of the results.

The reads left after rRNA filtering were mapped against the *Bos taurus* genome, downloaded from the illumina web-site (build UMD_3.1, version 75) (Zimin *et al.* 2009) using the TopHat2 (Kim *et al.* 2013) software with default parameters. The reads not mapping on the cow genome were collected and resynchronized by putting all the orphaned reads together with the unmapped single-end reads. Trinity (Grabherr *et al.* 2011) was used to assemble the resulting reads (strand specific option RF). For each transcript identified by Trinity, the Transdecoder software (Grabherr *et al.* 2011) was used to detect candidate coding regions (CDSs) using a minimum length threshold for the putative proteins of 80 amino acids and using the option to support the prediction with the Pfam–A and –B models. The final dataset of CDSs is composed by 56,343 protein sequences with an average length of 135.2 residues.

**References**

Andrews, S. FastQC. 2011.
Ref Type: Computer Program

Grabherr MG, Haas BJ, Yassour M, Levin JZ, Thompson DA, Amit I, Adiconis X, Fan L, Raychowdhury R, Zeng Q, Chen Z, Mauceli E, Hacohen N, Gnirke A, Rhind N, di PF, Birren BW, Nusbaum C, Lindblad-Toh K, Friedman N, Regev A (2011) Full-length transcriptome assembly from RNA-Seq data without a reference genome. *Nat.Biotechnol.* **29,** 644-652.

Kim D, Pertea G, Trapnell C, Pimentel H, Kelley R, Salzberg SL (2013) TopHat2: accurate alignment of transcriptomes in the presence of insertions, deletions and gene fusions. *Genome Biol.* **14,** R36.

Kopylova E, Noe L, Touzet H (2012) SortMeRNA: fast and accurate filtering of ribosomal RNAs in metatranscriptomic data. *Bioinformatics.* **28,** 3211-3217.

Magoc T, Salzberg SL (2011) FLASH: fast length adjustment of short reads to improve genome assemblies. *Bioinformatics.* **27,** 2957-2963.

Martin M (2011) Cutadapt removes adapter sequences from high-throughput sequencing reads. *EMBnet.journal* 10-12.

Quast C, Pruesse E, Yilmaz P, Gerken J, Schweer T, Yarza P, Peplies J, Glockner FO (2013) The SILVA ribosomal RNA gene database project: improved data processing and web-based tools. *Nucleic Acids Res.* **41,** D590-D596.

**Figure S1: Taxonomic composition of the samples.** The genus compositions of the Highly Expressed Core transcripts identified in the samples. The expression has been estimated as the sum of the reads mapped on transcript annotated to the taxonomic unit. Transcripts with less than 80% identity and 50% coverage with their best hit in Uniprot were not assigned to any genus. Unmapped reads and transcripts with no taxonomic annotation are not displayed.

**Figure S2: Bacterial vs. Antibody k-mer diversity.**

Bacterial (X axis) and antibody (Y axis) diversity display no significant correlation. Diversity is defined as the average percentage of unique k-mers for every 500,000 k-mers extracted from the bacterial and antibody reads. Error bars represent ±1 standard deviations, calculated on 10 random selections of 500,000 k-mers

**Table S3: Sequencing and Mapping summary**

| Sample | Raw | Processed |  |  | non rRNA | Bovine | Bacterial | Ratio |
| --- | --- | --- | --- | --- | --- | --- | --- | --- |
|  | Pairs | Total | Merged | Pairs | Total | Total | Total |  |
| Holm box11 | 65930116 | 56553812 | 55933014 | 620798 | 4449146 | 3587313 | 817316 | 4.44 |
| Holm box12 | 60651609 | 52263972 | 51100781 | 1163191 | 4415564 | 3585582 | 738158 | 4.98 |
| Holm box2 | 59658095 | 51888588 | 51250623 | 637965 | 4147425 | 3540066 | 559253 | 6.42 |
| Holm box3 | 58527507 | 50728838 | 49775990 | 952848 | 3603586 | 2934503 | 608621 | 4.92 |
| Holm box4 | 63552157 | 54232298 | 54099944 | 132354 | 4349764 | 3550949 | 788580 | 4.52 |
| Holm box7 | 44760148 | 38142899 | 37918712 | 224187 | 2661164 | 2284574 | 360871 | 6.37 |
| OrO box1 | 66748353 | 55406955 | 54031276 | 1375679 | 4056957 | 3313886 | 633606 | 5.40 |
| OrO box17 | 56084247 | 47802566 | 47152549 | 650017 | 4240608 | 3441030 | 744695 | 4.69 |
| OrO box19 | 60779863 | 52285276 | 51324489 | 960787 | 4163486 | 3342180 | 748606 | 4.56 |
| OrO box3 | 67027445 | 55331097 | 54392821 | 938276 | 4654692 | 3707653 | 865327 | 4.38 |
| OrO box4 | 61966054 | 51201278 | 50550779 | 650499 | 4643132 | 3727734 | 857223 | 4.42 |
| OrO box8 | 70564996 | 60773037 | 59501730 | 1271307 | 5079266 | 4081105 | 901004 | 4.64 |
| Ribe box2 | 54499458 | 46916461 | 45866916 | 1049545 | 3851158 | 3135798 | 637203 | 5.04 |
| Ribe box3 | 74437179 | 63341529 | 62691988 | 649541 | 5314774 | 4192809 | 1070750 | 3.96 |
| Ribe box4 | 55746823 | 47328910 | 46341783 | 987127 | 3772428 | 3118684 | 580356 | 5.50 |
| Ribe box5 | 63741677 | 52341239 | 51901898 | 439341 | 4673226 | 3791630 | 843333 | 4.54 |
| Slag box17 | 46190124 | 39772021 | 39661980 | 110041 | 3489845 | 2875213 | 606653 | 4.75 |
| Slag box22 | 41166439 | 35316424 | 34875167 | 441257 | 2711934 | 2296162 | 389268 | 5.97 |
| Slag box24 | 60307865 | 51581210 | 50745515 | 835695 | 4133946 | 3141625 | 929789 | 3.45 |
| Slag box9 | 59169309 | 51426942 | 51124472 | 302470 | 4313757 | 3591551 | 701862 | 5.15 |
| Slag box6 | 59930208 | 50881802 | 49766605 | 1115197 | 4219494 | 3389096 | 740903 | 4.70 |

The number of sequenced reads (Raw), of reads after pre preprocessing (Processed), rRNA filtering (non rRNA), host filtering (Bovine), and the number of non-bovine reads used for the transcript assembly (Bacterial) is reported for each sample. The ratio between bovine and bacterial reads is reported in the least column.
