# Supplementary material for: A novel approach to probe host-pathogen interactions of bovine digital dermatitis, a model of a complex polymicrobial infection
Source: BMC Genomics. 2016 Dec 1;17:987. doi: 10.1186/s12864-016-3341-7 (PMC5142292; doi:10.1186/s12864-016-3341-7)
Supplement: Supplementary file 3 — Known virulence factors in T. denticola and T. phagedenis. (DOCX 125 kb) [file 12864_2016_3341_MOESM3_ESM.docx]

**Table S1: Known virulence factors in T.denticola and T.phagedenis**

|  |  |  |
| --- | --- | --- |
| Gene | Organism | Function |
| gi\|42526515\|ref\|NP_971613.1\| | Treponema denticola | flagellar filament core protein |
| gi\|42526782\|ref\|NP_971880.1\| | Treponema denticola | oligopeptide/dipeptide ABC transporter peptide-binding protein |
| gi\|42526983\|ref\|NP_972081.1\| | Treponema denticola | flagellar filament core protein |
| gi\|42526985\|ref\|NP_972083.1\| | Treponema denticola | flagellar filament core protein |
| gi\|42527218\|ref\|NP_972316.1\| | Treponema denticola | flagellar filament outer layer protein |
| gi\|42527455\|ref\|NP_972553.1\| | Treponema denticola | membrane lipoprotein TmpC |
| sp\|Q73KK2\|MGLA_TREDE | Treponema denticola | Galactose/methyl galactoside import ATP-binding protein MglA |
| sp\|Q73Q16\|DNAK_TREDE | Treponema denticola | Chaperone protein DnaK |
| tr\|E7D028\|E7D028_TREDN | Treponema denticola | Factor H binding protein |
| tr\|F7ITJ4\|F7ITJ4_TREDE | Treponema denticola | Protease complex-associated polypeptide |
| tr\|F7IW68\|F7IW68_TREDE | Treponema denticola | Chemotaxis protein CheA |
| tr\|F7IWF6\|F7IWF6_TREDE | Treponema denticola | Major outer sheath protein |
| tr\|Q73KK1\|Q73KK1_TREDE | Treponema denticola | Galactose/glucose-binding lipoprotein |
| tr\|Q73KY1\|Q73KY1_TREDE | Treponema denticola | DnaJ domain protein |
| tr\|Q73L11\|Q73L11_TREDE | Treponema denticola | Hemin-binding protein B |
| tr\|Q73M41\|Q73M41_TREDE | Treponema denticola | Hemolysin |
| tr\|Q73M50\|Q73M50_TREDE | Treponema denticola | Leucine Rich Repeat domain protein |
| tr\|Q73MP0\|Q73MP0_TREDE | Treponema denticola | Glycoprotease family protein |
| tr\|Q73MU9\|Q73MU9_TREDE | Treponema denticola | Flagellar filament outer layer protein FlaA, putative |
| tr\|Q73NT1\|Q73NT1_TREDE | Treponema denticola | Peptide ABC transporter, peptide-binding protein OppA |
| tr\|Q73NZ3\|Q73NZ3_TREDE | Treponema denticola | Flagellar basal-body rod protein FlgG |
| tr\|Q73PF7\|Q73PF7_TREDE | Treponema denticola | Cytoplasmic filament protein A |
| tr\|Q73Q50\|Q73Q50_TREDE | Treponema denticola | Cysteine protease domain, YopT-type |
| tr\|Q73QH2\|Q73QH2_TREDE | Treponema denticola | BNR domain protein |
| tr\|Q73QU8\|Q73QU8_TREDE | Treponema denticola | Methyl-accepting chemotaxis protein DmcB |
| gi\|488783938\|ref\|WP_002696345.1\| | Treponema phagedenis | D-ribose transporter ATP binding protein |
| gi\|488784537\|ref\|WP_002696944.1\| | Treponema phagedenis | chaperone protein DnaK |
| gi\|488784616\|ref\|WP_002697023.1\| | Treponema phagedenis | type I restriction modification DNA specificity domain protein |
| gi\|488784942\|ref\|WP_002697349.1\| | Treponema phagedenis | membrane protein |
| gi\|488785944\|ref\|WP_002698351.1\| | Treponema phagedenis | cytochrome C oxidase subunit II |
| gi\|488786530\|ref\|WP_002698937.1\| | Treponema phagedenis | hemin receptor |
| gi\|488786772\|ref\|WP_002699179.1\| | Treponema phagedenis | hypothetical protein |
| gi\|488787305\|ref\|WP_002699712.1\| | Treponema phagedenis | ABC transporter substrate-binding protein |
| gi\|488788700\|ref\|WP_002701107.1\| | Treponema phagedenis | hypothetical protein |
| gi\|490387077\|ref\|WP_004266532.1\| | Treponema phagedenis | flagellin |
| gi\|639865854\|ref\|WP_024752085.1\| | Treponema phagedenis | aminotransferase |
| gi\|639866081\|ref\|WP_024752140.1\| | Treponema phagedenis | chemotaxis protein |
| gi\|639866270\|ref\|WP_024752182.1\| | Treponema phagedenis | hypothetical protein |
| gi\|639866293\|ref\|WP_024752187.1\| | Treponema phagedenis | flagellin |
| gi\|639866321\|ref\|WP_024752193.1\| | Treponema phagedenis | chemotaxis protein CheA |
| gi\|639866505\|ref\|WP_024752234.1\| | Treponema phagedenis | sugar-binding lipoprotein |
| gi\|639866852\|ref\|WP_024752314.1\| | Treponema phagedenis | flagellar protein |
| gi\|639867489\|ref\|WP_024752459.1\| | Treponema phagedenis | flagellar basal body rod protein FlgG |
| gi\|639867986\|ref\|WP_024752585.1\| | Treponema phagedenis | hypothetical protein |
| gi\|639868018\|ref\|WP_024752593.1\| | Treponema phagedenis | flagellin |
| gi\|639869893\|ref\|WP_024753002.1\| | Treponema phagedenis | hypothetical protein |
| gi\|639869940\|ref\|WP_024753016.1\| | Treponema phagedenis | hypothetical protein |
| gi\|639870240\|ref\|WP_024753105.1\| | Treponema phagedenis | hypothetical protein |
| gi\|639870683\|ref\|WP_024753204.1\| | Treponema phagedenis | dihydrouridine synthase |
| gi\|639871077\|ref\|WP_024753288.1\| | Treponema phagedenis | membrane protein |
| gi\|639871799\|ref\|WP_024753445.1\| | Treponema phagedenis | hypothetical protein |
|  |  |  |
